# CRM1 Inhibitors for Antiviral Therapy

**DOI:** 10.3389/fmicb.2017.01171

**Published:** 2017-06-28

**Authors:** Cynthia Mathew, Reena Ghildyal

**Affiliations:** Respiratory Virology Group, Centre for Research in Therapeutic Solutions, Health Research Institute, University of CanberraCanberra, ACT, Australia

**Keywords:** CRM1, CRM1 inhibitors, CRM1-mediated nuclear export, CRM1 in cancer, CRM1-mediated export of viral proteins

## Abstract

Infectious diseases are a major global concern and despite major advancements in medical research, still cause significant morbidity and mortality. Progress in antiviral therapy is particularly hindered by appearance of mutants capable of overcoming the effects of drugs targeting viral components. Alternatively, development of drugs targeting host proteins essential for completion of viral lifecycle holds potential as a viable strategy for antiviral therapy. Nucleocytoplasmic trafficking pathways in particular are involved in several pathological conditions including cancer and viral infections, where hijacking or alteration of function of key transporter proteins, such as Chromosome Region Maintenance1 (CRM1) is observed. Overexpression of CRM1-mediated nuclear export is evident in several solid and hematological malignancies. Interestingly, CRM1-mediated nuclear export of viral components is crucial in various stages of the viral lifecycle and assembly. This review summarizes the role of CRM1 in cancer and selected viruses. Leptomycin B (LMB) is the prototypical inhibitor of CRM1 potent against various cancer cell lines overexpressing CRM1 and in limiting viral infections at nanomolar concentrations *in vitro*. However, the irreversible shutdown of nuclear export results in high cytotoxicity and limited efficacy *in vivo*. This has prompted search for synthetic and natural CRM1 inhibitors that can potentially be developed as broadly active antivirals, some of which are summarized in this review.

## Introduction

### Overview

Despite concerted effort, spearheaded by the World Health Organization (WHO), the number of deaths caused by infectious diseases is falling slowly. In 1990, an estimated 13 million people died from communicable diseases, including viral, bacterial, parasitic and nematode infections. There has been only a marginal drop in number of deaths from infectious disease in 2015 and is forecast to be around 13 million even in 2030 (WHO, 2011). Clearly, there remains a need for continuing drug development.

A major stumbling block in antiviral therapy is the emergence of escape mutants that frequently follows strategies that target viral components (Endy and Yin, 2000). A better, more effective, strategy would be to target an essential host protein that is exploited by diverse viruses; however, this has the associated risk of high cytotoxicity. The challenge is to develop a drug that is effective against numerous viruses, including emerging and re-emerging viruses, with minimal cytotoxicity (Hung and Link, 2011; Bekerman and Einav, 2015).

Regulated, appropriate translocation and subcellular localization of proteins is essential for regulation of replication, transcription, and translation (Weis, 2003). Dysregulation of this system is observed in several pathological conditions including cancer and viral infections (Weis, 2003; Hung and Link, 2011; Le Sage and Mouland, 2013). Chromosome Region Maintenance1 (CRM1), a nuclear transporter protein, mediates the export of around 220 proteins and mRNA across the nuclear envelope (NE), and is involved in regulation of processes involved with proliferation including cell cycle progression and apoptosis (Xu et al., 2012). CRM1 is a key protein overexpressed in several solid and hematological malignancies where mislocalization of tumor suppressor proteins promotes malignancy and tumor progression (Hung and Link, 2011). CRM1 is also utilized by viruses at various stages of their lifecycle to mislocalize cellular proteins as well as ensure appropriate localization of viral proteins (Le Sage and Mouland, 2013). Given that viruses of diverse families, e.g., retroviruses, DNA and RNA viruses exploit or modulate CRM1-mediated nuclear export, effective targeting of CRM1 would lead to a broadly effective drug potentially active against current and future virus infections.

## Nucleocytoplasmic transport

Spatial partition of the nucleoplasm from the cytoplasm by the NE in the eukaryotic cell allows cellular functions to be restricted to specialized organelles and enables a multi-layered functional regulation of fundamental cellular processes, such as DNA synthesis, RNA transcription/transport, protein translation/maturation, cell division and signal transduction (reviewed in Weis, 2003; Talamas and Capelson, 2015). The double membrane structure of the NE contains numerous nuclear pore complexes (NPC) that are the only conduit of macromolecular trafficking between the nucleus and the cytoplasm. Consisting of a central core structure with nuclear and cytoplasmic extensions, the NPC is a highly selective molecular sieve that regulates bidirectional transport of macromolecules larger than 60 kDa (Mattaj and Englmeier, 1998; Gorlich and Kutay, 1999). Although passive flux of molecules <55 kDa can occur, most transport through the NPC is mediated by members of the karyopherin superfamily, which recognize nuclear localization sequences (NLSs) or nuclear export sequences (NESs) on cargo molecules for transport into and out of the nucleus, respectively. Shuttling transport receptors mediate all cargo transport and operate via similar mechanisms. Initiated by the recognition of the signaling motifs (NLS or NES) on cargo molecules, the shuttling transport receptors, such as importins and exportins, carry out nucleocytoplasmic exchange from the originating compartment to the target destination. Upon delivery, the empty receptors cycle back to mediate additional rounds of transport (Gorlich and Mattaj, 1996; Mattaj and Englmeier, 1998; Khmelinskii et al., 2014).

### Nuclear import

Nuclear import includes sequential docking/undocking events between the cargo/importin complex and the membrane-imbedded NPC, followed by the disassociation of the complex in the nucleoplasm, where the binding of RanGTP to importin releases it from the transport complex (Gorlich and Mattaj, 1996; Macara, 2001; Terry et al., 2007; Chumakov and Prassolov, 2010).

### Nuclear export

**Figure 1:**
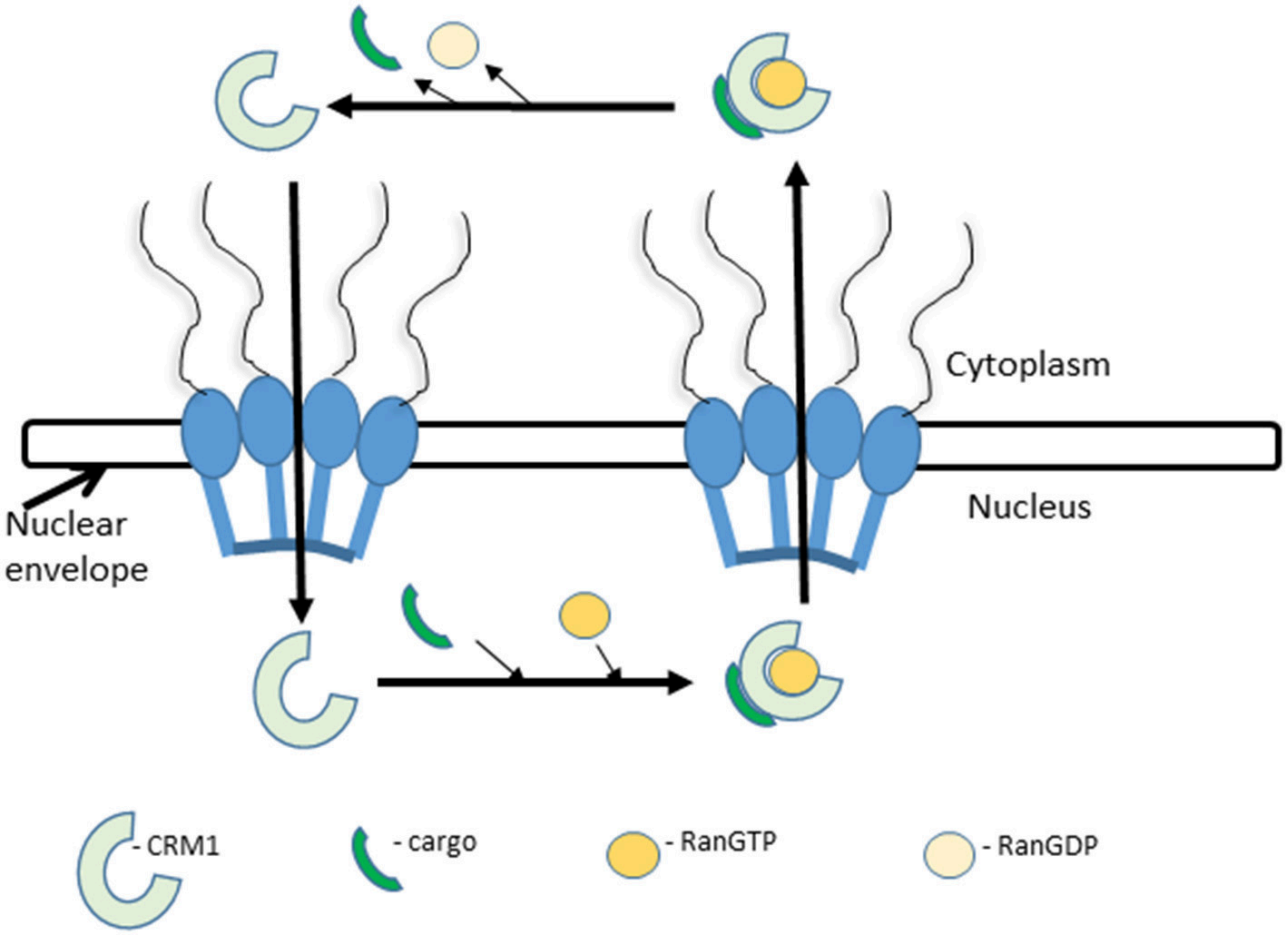
Nuclear export. Nuclear export is initiated by the recognition of an NES-carrying cargo by CRM1 and formation of a tricomplex with RanGTP. This is followed by sequential docking and undocking events at the NPC. After translocation into the cytoplasm the tricomplex is dissociated by the hydrolysis of RanGTP to RanGDP by RanGAP. The released CRM1 protein returns to the nucleus and repeats the process.

Nuclear export is essentially the same as nuclear import, in the opposite direction; with the main difference that RanGTP is an integral component of the complex in nuclear export, transporting the cargo/exportin from the nucleus to the cytoplasm (Figure 1). One of the most well characterized exportins is (CRM1; also, referred to as Exportin1 or XPO1). CRM1 binds to cargoes in the nucleus in the presence of RanGTP via a NES composed of a cluster of leucine (L)-rich or hydrophobic amino acids. After transit the hydrolysis of Ran-GTP by RanGAP (a GTPase) disassembles the trimeric complex and CRM1 re-enters the nucleus (Figure 1; Gorlich and Mattaj, 1996; Macara, 2001; Hutten and Kehlenbach, 2007; Chumakov and Prassolov, 2010; Xu et al., 2012).

## CRM1

Chromosome Region Maintenance1 (CRM1) architecture is conserved across species including humans, mice, fungi and yeast (Fung and Chook, 2014). CRM1 is a 120 kDa ring-shaped karyopherin protein composed of 21 tandem HEAT repeats, designated H1-H21, containing a pair of anti-parallel helices A and B that form the outer convex and inner concave surfaces (Dickmanns et al., 2015). The NES-binding groove on the surface of CRM1 is located between H11 and H12. CRM1 binds to protein cargoes at its outer convex surface by anchoring to key hydrophobic residues in the NES peptide into the NES-binding groove, unlike importins which bind with residues on their inner surface. Conformational changes involving H21 and H9 loop (a long conserved loop that connects H9A and H9B) are key structural elements crucial for CRM1 function. CRM1 adopts a ring-shaped structure with its N- and C-terminal HEAT repeats in close proximity which compacts further on forming a complex with the cargo and Ran GTP. In the unliganded state the H9 loop, which connects H9A and H9B, brings the helices H11A and H12A closer to each other and cuts off access to the NES groove. In addition, H21 crosses the CRM1 ring, interacts with both the H9 loop and the NES binding groove to render CRM1 inactive. In the CRM1-cargo-Ran GTP complex, H21 aligns itself with the CRM1 ring and the H9 loop adopts a beta-hairpin structure which displaces it from the NES binding groove. These conformational changes are crucial for the functioning of CRM1 whereby it adopts three conformations including an inactive ligand-free state with a closed NES groove, an active NES and RanGTP-bound state with an open groove and an intermediate NES bound (without RanGTP) state. The cysteine residue located within the hydrophobic NES-binding region at position 528 is the prime target for most CRM1 inhibitors including leptomycin B (LMB) (Petosa et al., 2004; Monecke et al., 2009; Sun et al., 2013; Fung and Chook, 2014; Turner et al., 2014).

### Functions

**Figure 2:**
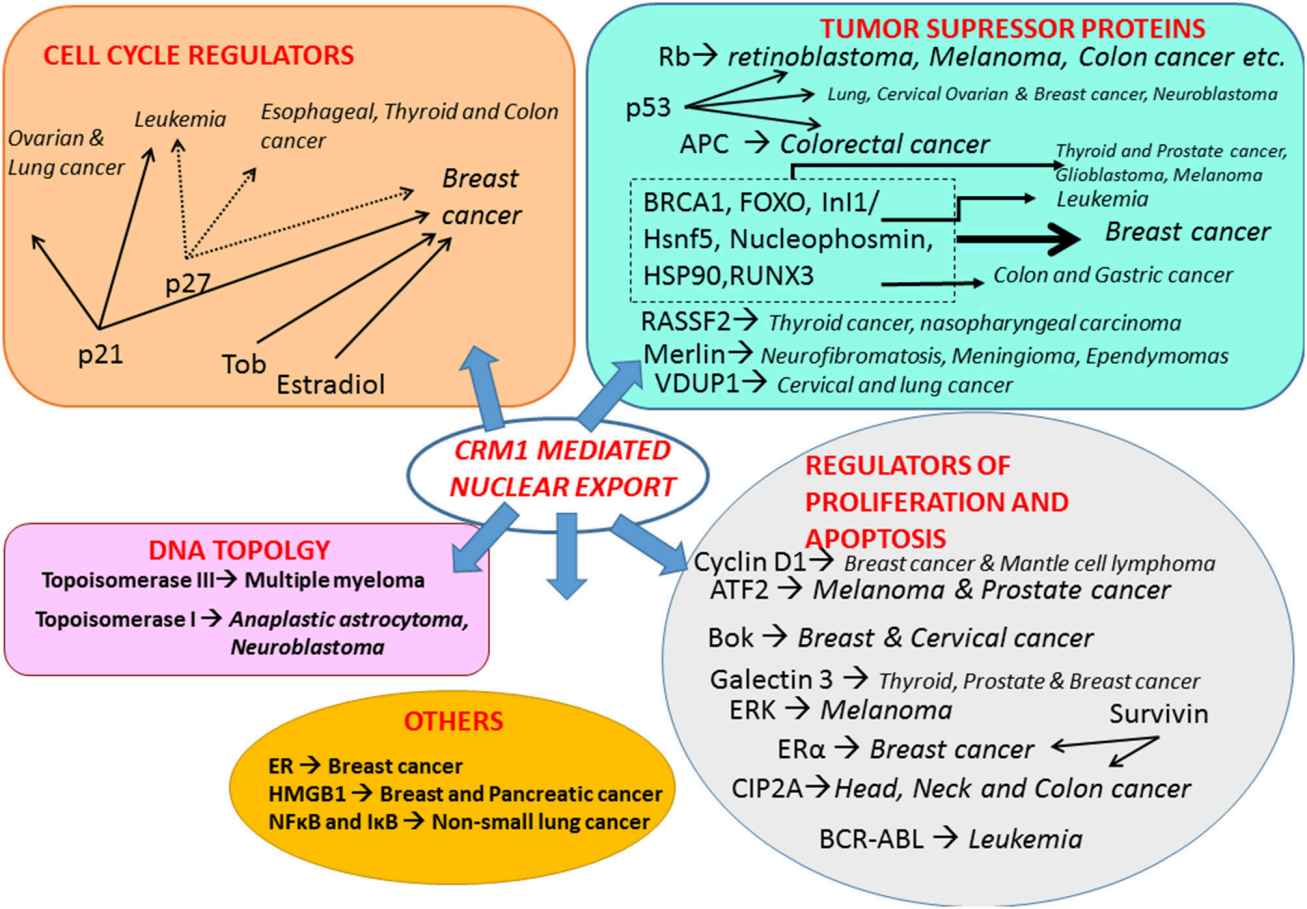
Function of CRM1-mediated export and its significance in cancer. The illustration summarizes some of the key proteins, including tumor suppressor proteins, cell cycle regulators, mediators of cell proliferation and apoptosis, proteins involved in maintenance of chromosomal and nuclear structures and others, regulated by CRM1-mediated nuclear export and their role in several solid and/or hematological malignancies. Abbreviations. APC, Adenomatous Polyposis Coli; ATF2, Activating transcription factor 2; BCR-ABL, Breakpoint Cluster Region/Abelson murine leukemia viral oncogene homolog 1 Bok, Bcl-2 related ovarian killer; BRCA1-Early Onset Breast Cancer 1; CIP2A, Cancerous Inhibitor of PP2A; ERα, Estrogen Receptor; ERK, Extracellular signal-Regulated Kinases; FOXO, Forkhead family of transcription factors; HMGB1, High Mobility Group Box 1; Hsp90, Heat Shock Protein 90; RASSF2, Ras association (RalGDS/AF-6) domain family member 2; RB, Retinoblastoma; RUNX3, Runt-related transcription factor 3; Tob, Transducer of ErbB-2.

Among the seven known nuclear export proteins in the Karyopherin family, CRM1 is the best characterized nuclear exporter. CRM1 is the sole nuclear exporter of several cellular growth and survival factors including proteins and RNA. CRM1-mediated transport mediates cell proliferation through several pathways (Figure 2). (i) The subcellular localization of NES-containing oncogenes and tumor suppressor proteins involved in regulating cell division, controlling apoptotic pathways, and maintain genomic integrity by recognizing and repairing DNA damage. Many of these regulatory proteins are mislocalized in a large variety of tumors (Turner et al., 2014; Sun et al., 2016). (ii) The control of mitotic apparatus and chromosome segregation through the regulated export of centrosome-associated proteins. For instance, inhibition of nucelophosmin by LMB results in its dissociation from centrosomes and premature centrosome duplication (Wang et al., 2005). BRCA1, a tumor suppressor protein commonly mutated in breast and ovarian cancer, stimulates DNA repair at the nucleus and inhibits centrosomal duplication in response to DNA damage (Arnaoutov et al., 2005; Nguyen et al., 2012). Disruption of CRM1-mediated export blocks the localization of BRCA1 at the centrosome and results in failure for cells to detect DNA damage (Brodie and Henderson, 2012). (iii) *crm1* yeast mutants had altered chromosomal structures that appeared as rod-like thickened fibers suggesting a role for CRM1 in maintenance of chromosomal and nuclear structures (Toda et al., 1992). In addition, abnormal nuclear morphology and cell cycle arrest at both G1 and G2 phases were observed in leptomycin-treated yeast (Nishi et al., 1994). CRM1 levels remain constant throughout the cell cycle and it is mainly localized to the NE in highly specialized cellular bodies called CRM1 nuclear bodies (CNoBs) that depend on RNA polymerase1 activity, suggesting a role in ribosome biogenesis (Gravina et al., 2014).

The structure and functions of CRM1 are dealt with in detail in several excellent reviews and will not be discussed further in this review.

## CRM1 in cancer

Shuttling regulatory proteins into and out of the nucleus is essential for regulation of cell cycle and proliferation. Cancer cells utilize nucleocytoplasmic trafficking pathways to stimulate tumor growth and to evade apoptosis (Gravina et al., 2014). There are numerous studies showing that protein up-regulation, or RNA/DNA amplification of importin and/or CRM1, correlates with neoplasia and poor prognosis (Senapedis et al., 2014). CRM1 is the sole nuclear exporter of several tumor supressor proteins and growth regulatory proteins including p53, p21, p73, Rb1, Adenomatous polyposis coli (APC), BCR-ABL, FOXO, and STAT3 (Parikh et al., 2014; Turner et al., 2014; Sun et al., 2016). Nuclear export of tumor suppressor proteins in normal cells prevents them from interacting with transcription factors in the absence of DNA damage or oncogenic stimuli (Parikh et al., 2014).

Overexpression of CRM1 is observed in solid and hematologic malignancies (Turner and Sullivan, 2008; Parikh et al., 2014; Das et al., 2015). Overexpression of CRM1 results in mislocalization of regulatory factors away from their original site of action in the nucleus and disrupts DNA topology, tumor suppression, cell cycle, and apoptosis (Turner et al., 2012a). This promotes malignancy, evasion of apoptosis and immune detection, and develops drug resistance.

Mutations in tumor suppressor proteins also result in mislocalization as it disrupts its ability to bind to CRM1 and exit the nucleus for proteosomal degradation. Overexpression of CRM1 in cervical cancer cell lines reduced the nuclear retention of several tumor suppressors including p53, p27, p21, and p18. siRNA-induced inhibition of CRM1 in cervical cancer cell lines significantly reduced proliferation and promoted cell death, while non-cancer cells remained unaffected (van der Watt et al., 2009).

Mutations in some cancer-associated proteins produce truncated products lacking NES or with reduced capability to bind to CRM1, resulting in increased nuclear retention (Lu et al., 2015). For instance, APC is a tumor suppressor protein that regulates β-catenin, a major component of the Wnt signaling pathway, and suppresses tumor progression. In a normal cell, APC chaperones β-catenin and promotes its CRM1-mediated export into the cytoplasm where β-catenin level is regulated by degradation. Mutations in APC gene cause malignant colon cancer and the intestinal polyp disorder familial adenomatous polyposis (Powell et al., 1992). The mutated APC accumulates in the nucleus, becomes less efficient in binding to β-catenin and retards CRM1-mediated export thereby promoting oncogenic cellular transformation (Powell et al., 1992; Henderson, 2000).

Chromosome Region Maintenance1 (CRM1) is therefore a promising cancer drug target, and the use of small molecule inhibitors of CRM1 for a variety of cancers has been reviewed in detail (Turner et al., 2012a, 2014; Gravina et al., 2014; Parikh et al., 2014; Senapedis et al., 2014; Tan et al., 2014; Das et al., 2015) and will not be discussed further.

## CRM1 in viral infections

Chromosome Region Maintenance1 (CRM1) has a key role in viruses from diverse families, including retroviruses, orthomyxoviruses, paramyxoviruses, flaviviruses, coronaviruses, rhabdoviruses, and herpesviruses. CRM1-mediated export is co-opted by many viruses during various stages of the viral lifecycle. Interruption of CRM1-mediated export results in changes in virion protein expression, virion replication, incomplete viral assembly, reduced infectivity, and improved antiviral host immune responses (Elton et al., 2001; Pasdeloup et al., 2005; Cao and Liu, 2007; Sanchez et al., 2007; Ghildyal et al., 2009; Cao et al., 2012; Liu et al., 2012; Nakano and Watanabe, 2016). In the following sections, we review the utilization of CRM1 and its role in the lifecycle of representative viruses from selected families.

### Human immunodeficiency virus type 1 (HIV-1)

A member of the family *Retroviridae*, HIV-1 encodes nine genes arranged as a series of 12 alternatively spliced exons (Kimura et al., 1996). Retroviral replication requires translation of fully spliced mRNA encoding Tat, Rev, and Nef proteins early in infection followed by cytoplasmic expression of a set of late-phase unspliced or partly spliced mRNAs encoding structural and accessory proteins (Kimura et al., 1996; Najera et al., 1999). HIV Rev protein is a 19 kDa phosphoprotein that mediates controlled expression of 4 and 9 kb retroviral mRNAs (encoding the *vif*, vpr, and *vpu*/*env* genes and the *gag*/*pol* gene, respectively) in the nucleus (Najera et al., 1999; Fontoura et al., 2005). Rev protein carries an NLS peptide enabling translocation into the nucleus, an RRE-binding (Rev response element) domain that binds to the unspliced mRNA, as well as an “activation” domain which contains the NES peptide which allows shuttling of the transcripts into the cytoplasm using CRM1-mediated export (Table 1; Kimura et al., 1996; Wolff et al., 1997; Najera et al., 1999; Urcuqui-Inchima et al., 2011). The binding of Rev to unspliced transcripts carrying the RRE creates an RNP filament with the NES displayed on the surface and provides a transient “tag” which directs to CRM1 (Najera et al., 1999).

**Table 1:** Role of CRM1-mediated nuclear export in viral lifecycles.

**Virus**	**Family**	**Genome**	**Viral protein**	**Effect of LMB**	**Viral protein function**	**References**
Human immunodeficiency virus type 1 (HIV-1)	*Retroviridae*	ssRNA	Rev	Reduced Rev-dependent trafficking and had an inhibitory effect on virus assembly, packaging and budding.	Translocation of unspliced and partially spliced mRNA.	Cao and Liu, 2007; Urcuqui-Inchima et al., 2011; Perwitasari et al., 2014
Human T-cell leukemia virus type-1 (HTLV-1)	*Retroviridae*	ssRNA	Rex	Inhibits Rex-mediated export of tax/rex RNA. Absence of Tax and Rex proteins causes HTLV-1 infection to be abortive and reduce viral titre.	Translocation of Rex-viral mRNA complex.	Younis and Green, 2005; Nakano and Watanabe, 2016
Influenza	*Orthomyxoviridae*	(−)ssRNA	Nuclear export protein (NEP)	Nuclear retention of vRNP. Reduces lung influenza virus burden. Decreased proinflammatory cytokine expression.	Mediates translocation of viral ribonucleoproteins (vRNP) into the cytoplasm.	Elton et al., 2001; Boulo et al., 2007; Watanabe et al., 2011; Perwitasari et al., 2014
Respiratory Syncytial Virus (RSV)	*Peumoviridae*	(−)ssRNA	Matrix protein	Interferes with its interaction with viral components in the cytoplasm and disrupts virus assembly.	Host genome silencing, viral assembly and budding.	Ghildyal et al., 2006, 2009, 2012
				Reduces viral titer.		
Dengue Virus	*Flaviviridae*	(+)ssRNA	NS5 protein	Altered virus production and decreased expression of antiviral chemokine IL8.	Modulation of host antiviral responses, particularly IL-8 induction, and viral replication.	Pryor et al., 2006; Rawlinson et al., 2009
Rabies Virus	*Rhabdoviridae*	(−)ssRNA	P protein	Disrupts viral assembly. Reduced pathogenicity due to loss of capability to evade host antiviral interferon responses.	Inhibits both type I and II IFN responses, enables stable viral replication and determines neuroinvasion.	Gupta et al., 2000; Pasdeloup et al., 2005; Vidy et al., 2007
Human cytomegalovirus (HCMV)	*Herpesviridae*	dsDNA	Matrix protein phosphoprotein 65 (pp65)	Retards viral replication.	Phosphorylates the viral immediate-early proteins and protects them from being recognized by the host immune system.	Abate et al., 2004; Sanchez et al., 2007
					Retards the expression of both MHC class 1 and II molecules thus crippling viral recognition by both CD4 and CD8 T Helper cells.	

HIV-1 Rev is an indispensable regulatory factor for virion protein expression (Cao and Liu, 2007). The unspliced and partially spliced mRNA will be degraded in the absence of Rev, resulting in interruption of HIV-1 replication at the same time (Cao and Liu, 2007). CRM1-assisted export of gag mRNA enables efficient processing of Gag proteins and production of viral particles (Nagai-Fukataki et al., 2011). Disruption of the Rev RRE-CRM1 complex and inhibition of CRM1/Rev-mediated viral RNA transport using CRM1 inhibitors, such as LMB, ratjadone A (Fleta-Soriano et al., 2014), and a synthetic low molecular weight compound PKF050-638 (Daelemans et al., 2002) arrests transcription of HIV-1, inhibits the production of new virions and reduces HIV-1 levels (Urcuqui-Inchima et al., 2011; Perwitasari et al., 2014).

### Human T-cell leukemia virus type-1 (HTLV-1)

Belonging to the genus *Deltaretrovirus* of the *Retroviridae* family, HTLV-1 causes adult T-cell leukemia, HTLV-1 associated myelopathy/tropical spastic paraparesis and HTLV-1 uveitis. After HTLV-1 entry, the viral genomic RNA is reverse-transcribed and integrated into the host genome. HTLV-1 genomic RNA encodes more than 10 viral proteins and has three alternatively-spliced forms of viral mRNAs that are unspliced (encodes Gag, Pro, and Pol proteins), singly (partially)-spliced (encodes Env) and doubly (fully)-spliced (encodes accessory proteins, such as Tax, Rex, P30II, p12, and p13). The viral mRNA from the provirus for the first round of transcription is completely spliced to tax/rex mRNA by the cellular splicing machinery. Tax stimulates gradual accumulation of Rex in the nucleus which in turn permits Rex-mediated nuclear export of unspliced and partially spliced viral RNA into the cytoplasm (Younis and Green, 2005; Knipe and Howley, 2013; Nakano and Watanabe, 2016).

Similar to the HIV-1 Rev protein, HTLV-1 Rex protein recognizes the Rex Responsive Element (RxRE) on the mRNAs to form a Rex-viral mRNA complex for selective nuclear-export using CRM1 (Table 1) (Nakano and Watanabe, 2016). Unlike the RRE in HIV-1 mRNAs, RxRE is in all HTLV-1 mRNAs but they differ in nuclear export efficiency by Rex (Nakano and Watanabe, 2016). Rex is indispensable for efficient viral replication, infection and spread since it is considered to regulate the switch between the latent and productive phases of the HTLV lifecycle (Younis and Green, 2005). Without a functional Rex, viral structural and enzymatic post-transcriptional gene expression would be severely repressed and lead to non-productive viral replication (Younis and Green, 2005).

### Influenza

The family *Orthomyxoviridae* consists of negative sense, single-stranded, segmented RNA viruses, influenza being the prototypic virus. The replication cycle of orthomyxoviruses consist of attachment, receptor-mediated endocytosis followed by release of the viral ribonucleoprotein (vRNP) in the cytoplasm. The vRNP is then imported into the nucleus where it undergoes transcription and replication. The newly formed vRNPs are exported to the plasma membrane to complete assembly of viral particles and budding (Knipe and Howley, 2013) The RNA genome of influenza virus encodes 11 viral proteins. The transmembrane proteins hemagglutinin, neuraminidase and M2 protein on the viral envelope assist in attachment and penetration, as well as M2 protein-mediated release of the vRNP into the cytoplasm. The vRNP consists of the (−)ssRNA, three polymerase proteins (PB1, PB2, and PBA) that form the viral RNA polymerase complex (3P) and nucleoprotein (NP). Nuclear import of the vRNP is facilitated using importin-α (Imp-α). Following transcription and replication in the nucleus, newly synthesized RNA is complexed with NP to form new vRNPs; reviewed in Nayak et al. (2004) and Boulo et al. (2007).

Transport of the vRNP into the cytoplasm is coordinated by the NES-containing viral nuclear export protein (NEP). NEP, formally called NS2, utilizes the CRM1-mediated nuclear export pathway to transport the viral core to the assembly site at the plasma membrane (Table 1; Elton et al., 2001; Boulo et al., 2007; Perwitasari et al., 2014). This is a critical step for the formation of mature viral particles and is conserved in influenza A, B and C strains (Paragas et al., 2001; Paterson and Fodor, 2012). In addition to NEP, the viral matrix protein (M1) and NP also play important roles in this step. Absence of either protein results in retention of vRNPs in the nucleus (Bui et al., 2000; Neumann et al., 2000). CRM1 silencing resulted in reduced viral replication, lung viral load and proinflammatory cytokine expression (Watanabe et al., 2001; Perwitasari et al., 2014).

### Respiratory syncytial virus (RSV)

Reclassified under the newly formed *Pneumoviridae* family, formerly under *Paramyxoviridae*, RSV is the major causative agent of lower respiratory tract infections among infants, immunocompromised and elderly with no approved antiviral therapy or vaccines (Simoes et al., 2015; Afonso et al., 2016). RSV consists of a nucleocapsid core, wherein the RNA genome is tightly encapsulated by nucleocapsid proteins and the viral polymerase complex, contained within a lipid bilayer envelope. RSV infection is initiated when the large glycoprotein G facilitates attachment of the virion to the host membrane followed by fusion of the viral envelope with the host membrane mediated by the viral F protein. The nucleocapsid core is then released into the cytoplasm where the virus-encoded polymerase complex formed by L and P proteins, direct the transcription of the (−)ssRNA genome to generate the primary mRNA transcripts, which are then translated into viral proteins (Ghildyal et al., 2006, 2009). The Matrix (M) protein possesses both NLS and NES sequences that enable it to shuttle in and out of the nucleus in the early and late stages of infection using importin β (Imp-β) and CRM1, respectively. At the early stages of viral replication [about 5–6 h post infection (h.p.i)], the newly-synthesized M protein translocates into the nucleus via the action of the nuclear transport protein Imp-β and accumulates in the nucleus up to 16 h.p.i (Ghildyal et al., 2006, 2009; Bajorek et al., 2014).

Specific inhibition of CRM1-mediated nuclear export by LMB increased nuclear accumulation of M protein and reduced virus production (Table 1). In addition, mutations in the CRM1 recognition site prevented successful nuclear export of M protein, which eventually halted virus production due to the failure of M protein to localize at the assembly sites. This shows that timely CRM1-dependent nuclear export of M protein is central to RSV infection (Ghildyal et al., 2006, 2009, 2012; Bajorek et al., 2014).

### Dengue virus

Dengue viruses are the major causative agents of arthropod-borne viral diseases, such as dengue fever and dengue hemorrhagic fever. A member of the *Flaviviridae* family, the dengue virus (+)ssRNA genome encodes ten viral proteins including three structural proteins (capsid, pre-membrane/membrane, and envelope) and seven non-structural (NS1, NS2A, NS2B, NS3, NS4A, NS4B, and NS5) proteins (Knipe and Howley, 2013).

Following adsorption, the virus is internalized via receptor-mediated endocytosis. The acidic pH of the endosome promotes fusion of the viral endosomal membranes and allow the release and uncoating of the viral nucleocapsid into the cytoplasm. The RNA genome is then translated and replicated in the cytoplasm. Viral assembly is conducted at the endoplasmic reticulum following several rounds of translation followed by maturation of the virion in the Golgi apparatus; reviewed in Clyde et al. (2006) and Knipe and Howley (2013).

The NS5 protein is the largest and highly conserved among dengue NS proteins. It localizes both in the cytoplasm and nucleus. The N terminal domain of NS5 carries out two biochemically distinct methylation reactions for RNA capping and the C terminal domain has an RNA-dependent RNA polymerase activity required for synthesis of the viral genome in the cytoplasm; reviewed in Lim et al. (2015). DENV-2 NS5 possesses two NLSs that are capable of binding Imp-α/β and Imp-β, respectively (Pryor et al., 2007). It also carries an NES sequence that interacts with CRM1 (Table 1; Pryor et al., 2006). The bidirectional transport of NS5 has been shown to be critical for modulation of host antiviral responses, particularly IL-8 induction, and viral replication. Inhibition of CRM1 in dengue virus type-2 infections resulted in nuclear accumulation of NS5, reduced IL-8 induction and altered viral replication kinetics (Pryor et al., 2006; Rawlinson et al., 2009).

### Rabies virus

*Rhabdoviridae* consist of a morphologically distinct group of enveloped ss(−)RNA viruses with an elongated rod-like or bullet-like shape. Rabies virus ss(−)RNA genome encodes five viral proteins and replicates entirely in the host cell cytoplasm. The nucleoprotein (N), the large protein (L) and the phosphoprotein (P) enclose the genome to form the virion core that is further enclosed by the matrix (M) protein followed by a viral envelope embedded with glycoprotein (G). L and P form an RNA-dependent RNA polymerase complex and are involved in viral transcription and replication. The rabies virus P protein also acts as a regulatory protein that antagonizes interferon-mediated antiviral responses of the host cell (Knipe and Howley, 2013).

The P protein is expressed as five isoforms P1 to P5. The nucleocytoplasmic localization of these P isoforms is dependent on the activity of the NES and NLS sequences located at the N- and C-terminals, respectively. P1 and P2 carry the dominant CRM1-dependent NES while P3–P5 carry a truncated version of NES allowing NLS to become the primary localization signal. This results in P1 and P2 being localized in the cytoplasm while P3–P5 are nuclear. (Table 1; Pasdeloup et al., 2005; Oksayan et al., 2012). NES of P protein is conserved throughout the *Lyssavirus* genus (Pasdeloup et al., 2005). P protein-mediated antagonism of interferon production in the host cell has been shown to play a major role in neuroinvasion and infection of peripheral nerves as it is essential for stable viral replication (Yamaoka et al., 2013). In normal cells, the phosphorylation of STAT1 enables it to localize in the nucleus and regulate gene expression. P protein inhibits the phosphorylation, nuclear translocation and DNA binding activity of STAT1 resulting in inhibition of both type I and II interferon responses (Vidy et al., 2007). In addition, P protein interferes with the phosphorylation of interferon regulatory factor 3 (IRF-3) to retard interferon production (Brzozka et al., 2005). Thus, P protein interaction is critical to rabies pathogenicity. Interplay between the NLS and NES sequences on P protein in combination with the phosphorylation and dephosphorylation of the signal peptides regulate the localization of the viral protein (Gupta et al., 2000; Pasdeloup et al., 2005). The complex interaction of P protein with the host cell trafficking machinery ensures success of infection and suppression of host antiviral responses (Oksayan et al., 2012).

### Human cytomegalovirus (HCMV)

*Herpesviruses* are highly disseminated in nature with hosts ranging from bivalves to humans. Named after their characteristic icosahedral architecture, a typical herpesvirus consists of an outer envelope, tegument or matrix layers, and a capsid enclosed dsDNA genome (Knipe and Howley, 2013). Human cytomegalovirus, the largest member of the herpesvirus family, is the most significant cause of congenital disease. The virion is composed of a glycoprotein embedded host-derived envelope that encloses the tegument layer and the viral core composed of the icosahedral protein capsid and a dsDNA genome. Viral gene expression takes place in three stages: immediate-early, early and late stages. The tegument (or matrix) layer, lies beneath the envelope and above the viral capsid, is composed of nearly 32 proteins that conduct a range of functions including host cell response and orchestrating viral assembly. These matrix proteins are added to the nucleocapsid partly in the nucleus and partly in the cytoplasm during viral maturation. In the early stages of infection, tegument proteins associate with microtubules and the NPC to deliver the viral genome into the nucleus. In the final stages of infection these proteins coordinate stabilization of the nucleocapsid, trafficking from the nucleus, assembly at the plasma membrane and budding (Kalejta, 2008; Knipe and Howley, 2013).

Many tegument proteins are conserved across herpesviruses and are highly immunogenic. The lower matrix protein phosphoprotein 65 (pp65) is the most abundant tegument protein and a prominent target for MHC class I–restricted CD8 and MHC class II CD4 T-cell responses (Frankenberg et al., 2012; Knipe and Howley, 2013). Pp65 localizes in the nucleus immediately after viral entry and carries out immune evasion tactics. Pp65 acts on two fronts, on one side it phosphorylates the viral immediate-early proteins and thereby protects them from being recognized by the host immune system (Gilbert et al., 1996). Pp65 also retards the expression of both MHC class 1 and II molecules thus crippling viral recognition by both CD4+ and CD8+ T cells (Odeberg et al., 2003; Frankenberg et al., 2012). Pp65 also retards the host interferon response, particularly type 1 interferon response, reducing expression of interferon beta and other cytokines (Abate et al., 2004). Disruption of CRM1-medited export of pp65 resulted in its nuclear retention and decreased viral replication (Sanchez et al., 2007). During the lytic stage of the viral cycle, pp65 utilizes CRM1-mediated transport to shuttle into the cytoplasm (Table 1; Sanchez et al., 2007; Frankenberg et al., 2012). Another key function of pp65 is to inhibit natural killer cell cytotoxicity by directly binding to the activation receptor NKp30 (Arnon et al., 2005).

Another tegument protein UL94 is a late protein carrying both NLS and CRM1-dependent NES sequences. Mutation in these sequences disrupted its intracellular localization and upset its shuttling function (Liu et al., 2012). Other tegument proteins play crucial roles during infection including transcription of the genome (pp71, ppUL35, ppUL69, UL47, and UL48), translocation of maturing nucleocapsids from the nucleus to the cytoplasm (pp150 and pp28), modifying host cell response to infection (pIRS1/pTRS1), interfering with host cell cycle (pp71, ppUL69, and UL97) and optimize the nuclear environment for viral replication; reviewed in Kalejta (2008) and Knipe and Howley (2013).

## Leptomycin B and synthetic inhibitors

Inhibition of nucleocytoplasmic transport by natural and synthetic products has been pursued as a therapeutic avenue in cancer based on a number of biologic observations (Niu et al., 2015). Several small molecule inhibitors of CRM1 have been developed and tested against a variety of neoplasms, primarily *in vitro*. These targeted therapies work, at least in part, by forcing nuclear accumulation of tumor suppressor proteins that are mislocalized or expressed at abnormal levels in cancer cells, to initiate cascades of apoptosis (Niu et al., 2015). The successful application of CRM1 inhibitors against cancer gives support to their potential as antiviral agents.

### Leptomycin B: the prototypical CRM1 inhibitor

Leptomycin B (LMB; also known as elactocin, mantuamycin, and NSC 364372) is a polyketide isolated from *Streptomyces* and the first specific inhibitor of CRM1 to be discovered (Mutka et al., 2009; Turner et al., 2012a). It rapidly induces cytotoxic effects in cancer cell lines via covalent inhibition of CRM1 (IC50 value 0.1–10 nM) (Mutka et al., 2009). Its nanomolar potency stems from a highly specific binding to Cys528 via a Michael-type addition and subsequent inhibition of CRM1-mediated export from the nucleus (Kudo et al., 1998, 1999). Its clinical development was discontinued midst a single phase 1 trial in mouse tumor models due to significant toxicity (anorexia and malaise) without apparent efficacy (Newlands et al., 1996).

#### Mechanism of action

**Figure 3:**
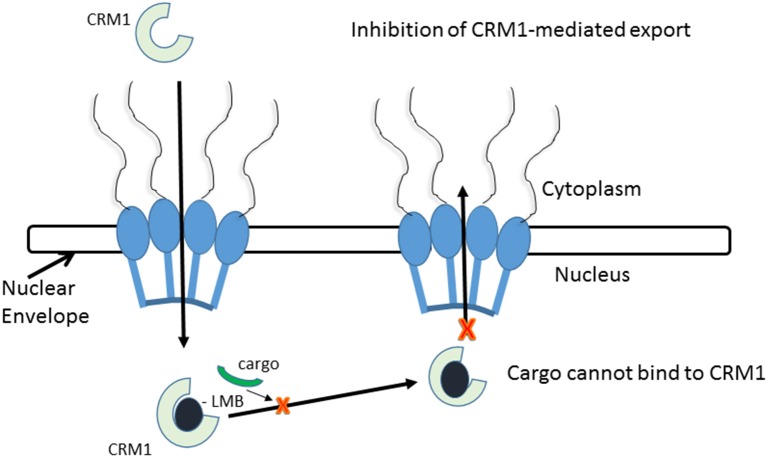
CRM1-inhibition by Leptomycin B. Leptomycin B binds to CRM1 at Cys528 residing in its NES-binding groove and inhibits the binding of the cargo to CRM1.

Leptomycin B (LMB) is chemically defined as 19-[(2S,3S)-3,6-dihydro-3-methyl-6-oxo-2H-pyran-2-yl]-17-ethyl-6-hydroxy-3,5S,7S,9R,11,15R,hexamethyl-8-oxo-2E,10E,12E,16Z,18E-nonadecapentaenoic acid. It is a polyketide with a α,β-unsaturated d-lactone portion that covalently binds through Michael addition to the Cys528 in the CRM1 NES groove (or Cys529 in *Saccharomyces pombe*). Binding with LMB initially forces CRM1 to remain in a half open conformation. This is followed by hydrolysis of the lactone group of LMB in the presence of basic amino acid residues in the NES groove and thereby stabilizes the interaction making it irreversibly bound to CRM1 (Figure 3; Kudo et al., 1998). The persistent shutdown of CRM1 mediated nuclear export and off target activity are possibly the reasons why it is highly deleterious to the cell (Newlands et al., 1996; Fung and Chook, 2014; Perwitasari et al., 2014). LMB has been shown to block the export of influenza viral ribonucleoproteins (vRNP) containing the (−)ssRNA genome, nucleoprotein and polymerases, from the nucleus into the cytoplasm and thereby inhibit influenza replication (Perwitasari et al., 2014). Table 1 lists some of the outcomes of LMB treatment in cells infected with selected viruses.

#### Side effects

Leptomycin B (LMB) binds covalently to Cys528 located in the NES-binding groove of CRM1, inactivates it in an irreversible manner. Hence, LMB is highly toxic and *in vivo* application is not justified (London et al., 2014). However, LMB alternatives ratjadone and anguinomycin A inhibit CRM1-mediated export almost identically, binding covalently to Cys528 with reduced cytotoxicity (Sun et al., 2013). This suggests that we can design/utilize synthetic and/or natural compounds inhibiting CRM1 in the same mechanism of action as LMB but with no or reduced cytotoxicity.

### Synthetic CRM1 inhibitors

#### Nuclear export inhibitors (NEIs)-KOS 2464

Mutka et al. have synthesized analogs of LMB with substantially improved therapeutic windows while maintaining the high potency of LMB. They had better *in vivo* tolerance, up to 16-folds higher than LMB (Table 2). Although the NEIs cause inhibition of CRM1 in normal and tumor cell types, the downstream consequences of this inhibition are different to that of LMB, inducing cell cycle arrest (instead of apoptosis) in normal lung fibroblasts and apoptosis in p53 wild-type cancer models (HCT-116 colon model and SiHa cervical cancer) (Mutka et al., 2009). Topoisomerase IIα (topo IIα) is a transcription factor that activates DNA-cleavable complexes and induces cell death. However, drug resistant myeloma cells mislocalize topo IIα to the cytoplasm using CRM1 nuclear export to escape apoptosis (Engel et al., 2004). KOS-2464 (Kosan Biosciences/Bristol-Myers Squibb), a semisynthetic NEI induces rapid and prolonged inhibition of CRM1, and apoptosis in drug resistant multiple myeloma cell lines at nanomolar concentrations (low IC50 value of 2 nM) without the toxicity associated with LMB (Turner et al., 2012b). Treated cells were found sensitive to topo IIα inhibitors while normal cells remained unaffected by the combined effects of CRM1 and topoIIα inhibition (Turner et al., 2009). In comparison to LMB, the absence of apoptosis despite cell cycle arrest by KOS2464 in normal lung fibroblasts is indicative of reduced off-target activity (Turner et al., 2012a).

**Table 2:** Inhibitors of CRM1 nuclear export.

**Drug**	**Source**	**Mechanism of action**	**Structure**	**IC 50**	**Effect on cells**	**References**
Leptomycin B (LMB)	*Streptomyces*	Binding to cysteine 528 located in the NES-binding groove	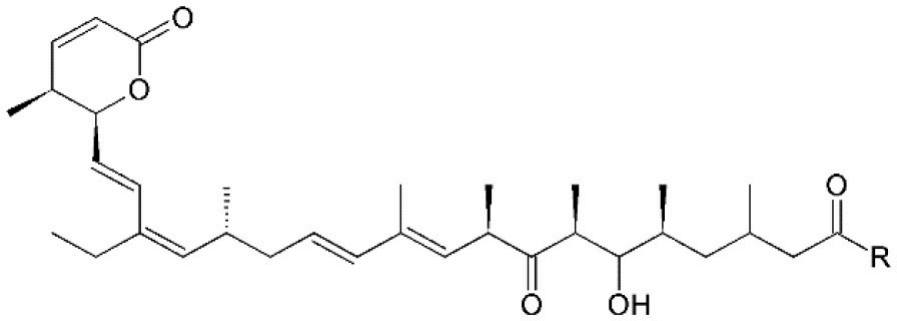	0.1–10 nM in tumor cell lines.	High efficacy *in vitro*.	Newlands et al., 1996; Wolff et al., 1997; Kudo et al., 1999; Mutka et al., 2009
					Inhibits Rev-dependent export of mRNA into the cytoplasm and retard HIV-1 replication.	
					Dose-limiting toxicity observed as anorexia and malaise in Phase1 clinical trials.	
Nuclear Export Inhibitors (NEIs), KOS-2464	Semi synthetic derivatives of LMB	Binding to cysteine 528 located in the NES-binding groove	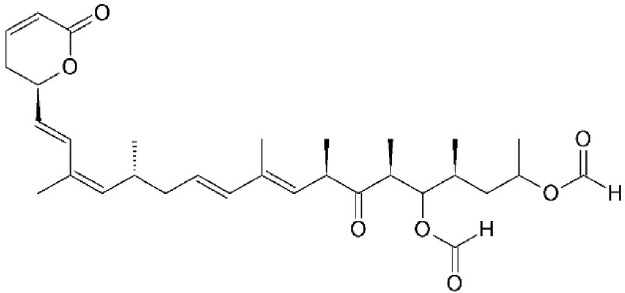	0.1–10 nM in tumor cell lines.	Rapid and prolonged block of CRM1-mediated nuclear export, results in apoptosis in tumor cell lines and cell cycle arrest (no apoptosis) in normal fibroblasts.	Mutka et al., 2009; Gravina et al., 2014
					16-fold less toxic than LMB *in vivo* mouse xenograft models.	
N-azolylacrylate analog, PKF050-638	Synthetic	Reversibly disrupts CRM1-NES interaction	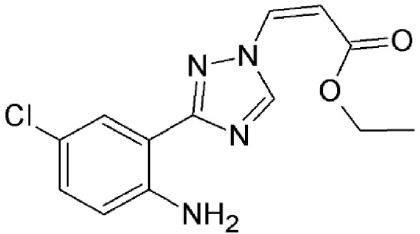	0.5 nM	Inhibits Rev-dependent luciferase gene expression in Jurkat cells.	Daelemans et al., 2002
CBS9106	Synthetic	Reversibly blocks CRM1–proteosome dependent degradation of CRM1	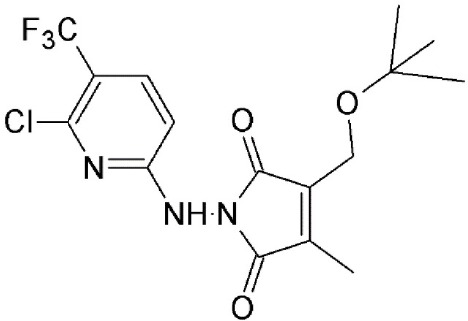	3–278 nM, value dependent on cell type.	Cell cycle arrest and induce apoptosis in a time and doe dependent manner across a broad spectrum of cancer cells.	Sakakibara et al., 2011; Saito et al., 2014
					Reduce NF-κB activity and promoted nuclear accumulation of tumor suppressor proteins including p53.	
S109	Synthetic derivative of CBS9106	Reversibly blocks CRM1–proteosome dependent degradation of CRM1	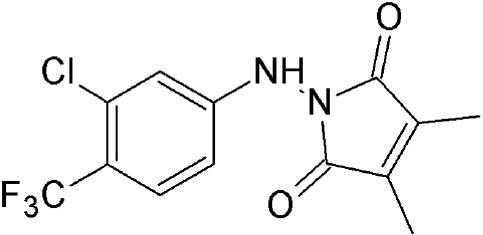	1.16 μM in ovarian cancer cells.	Reduce proliferation and colony formation in renal cancer cells.	Liu et al., 2016
					Cause cell cycle arrest at G1 phase, down regulate cyclinD1 and increase nuclear localization of p53, p21, p27 and FOXO1.	
**SINEs**						
Selinexor (KPT 330)	Synthetic	Covalently binds in a reversible manner to cysteine528	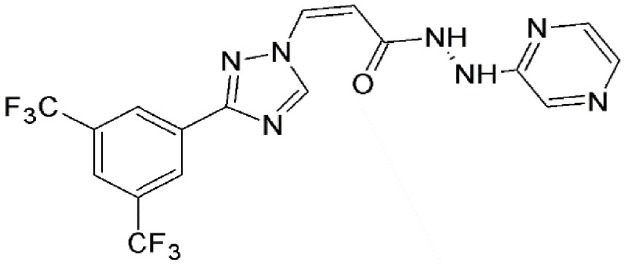	<500 nM	Specifically targets malignant cells. Dose dependent cytotoxicity in various solid and hematological malignancies.	Etchin et al., 2013; Salas Fragomeni et al., 2013; Gravina et al., 2014; Perwitasari et al., 2014; Tan et al., 2014; Zheng et al., 2014
KPT 185	Synthetic	Covalently binds in a reversible manner to cysteine528	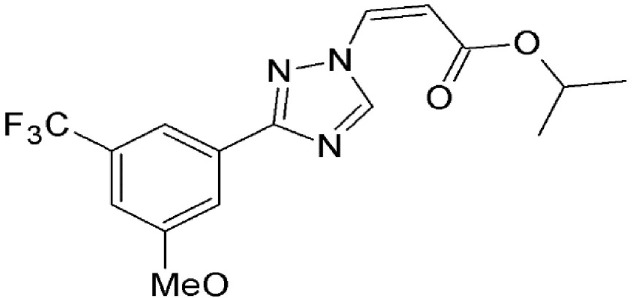		Induces cell cycle arrest and apoptosis in tumor cells. Increased nuclear localization of p53, p27, p73, Rb, FOXO1, APC, surviving etc.	
Verdinexor (KPT 335)	Synthetic	Covalently binds in a reversible manner to cysteine528	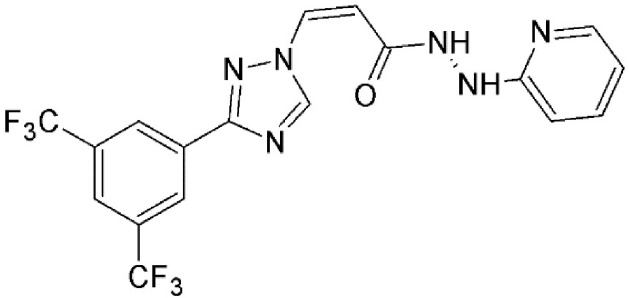		KPT 335 has been shown to inhibit nuclear export of viral RNP and reduce influenza replication.	
					Inhibits CRM1/Rev-mediated viral RNA transport and reduces HIV-1 replication.	
Valtrate	*Valeriana fauriei*	Covalently binds to cysteine528	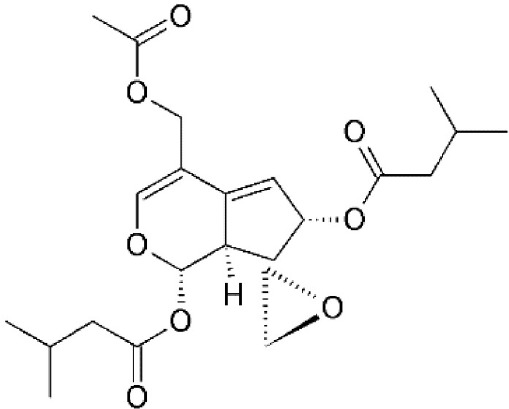	2.5 μM in HeLa cells.	Exhibits anti-HIV activity by inhibiting export of Rev protein from the nucleus to the cytoplasm.	Murakami et al., 2002; Tamura et al., 2010b; Watanabe et al., 2011
				0.19 μM in MDCK cells.	Exhibits anti-influenza activity by inhibiting nuclear export of vRNP.	
D,L1′-Acetoxychavicol acetate (ACA)	*Alpinia galangal*	Covalently binds to cysteine539	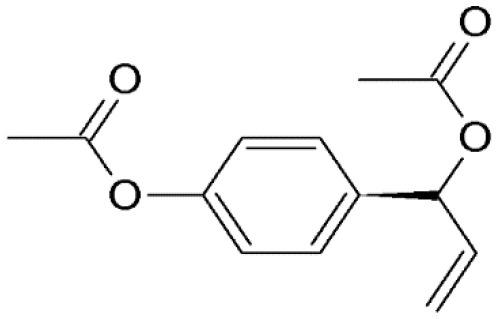	2.0 μM in MDCK cells.	Exhibits anti-influenza activity by inhibiting nuclear export of vRNP.	Watanabe et al., 2011
Prenylcoumarin osthol	*Cnidii Monnieris Fructus*	Covalently binds to cysteine528	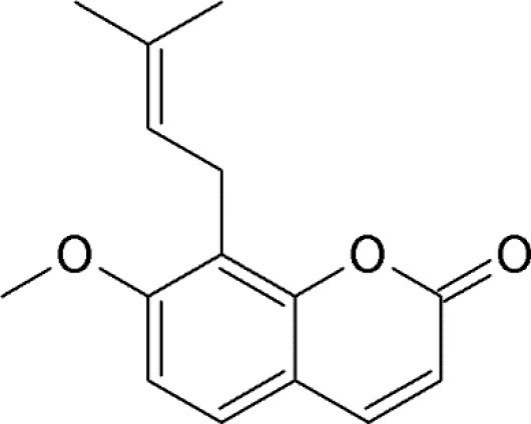	1.6 μM in HeLa cells.	Exhibits anti-HIV activity by inhibiting export of Rev protein from the nucleus to the cytoplasm.	Tamura et al., 2010a
Ratjadone (A-D)	*Sorangium cellulosum*	Covalently binds to cysteine528	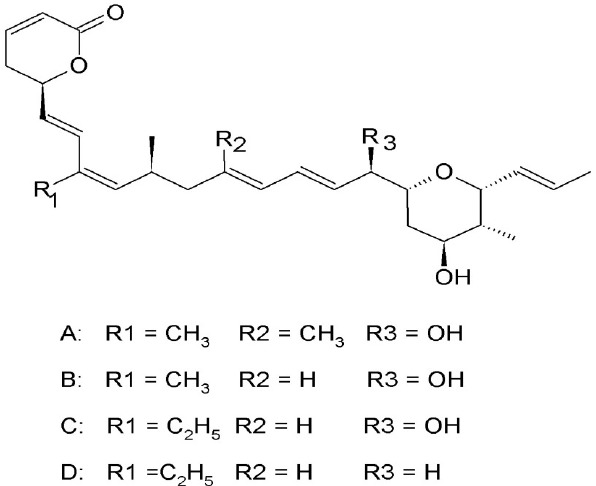	0.15–1 ng/ml.	Arrest cells at G1 phase.	Koster et al., 2003; Meissner et al., 2004
					Increases the size of nuclei.	
					Inhibits nuclear export of topoisomerase II α.	
	Semi-synthetic analogs					
Anguinomycins	*Streptomyces*	Covalently binds to cysteine528	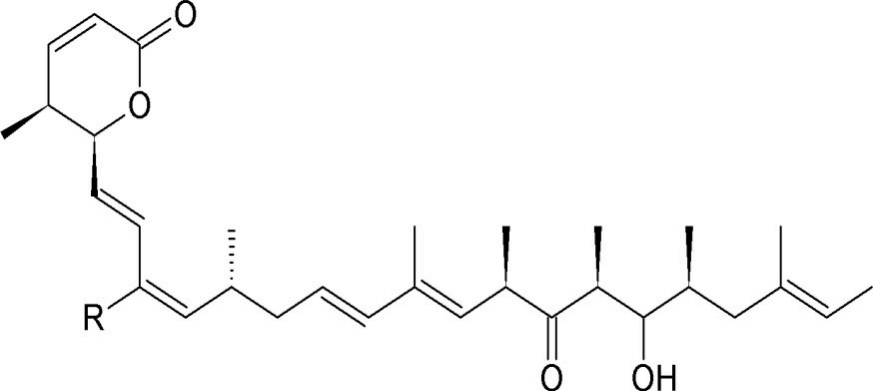	Inhibits CRM1 at concentrations >10 nM.	Selective toxicity against transformed cells at picomolar concentrations.	Bonazzi et al., 2010; Dickmanns et al., 2015
Goniothalamin	*Goniothalamus*	Covalently binds to cysteine528	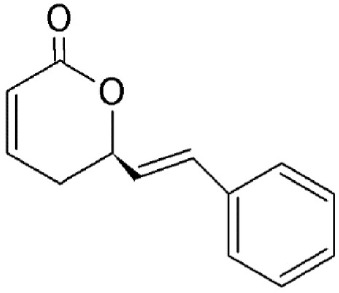	>500 nM *in vivo*.	Anti-proliferative and apoptotic effects in cancer cell lines. Upregulates p53 accumulation in the nucleus.	Chen et al., 2005; Wach et al., 2010
Piperlongumine	*Piper longum*	Covalently binds to cysteine528	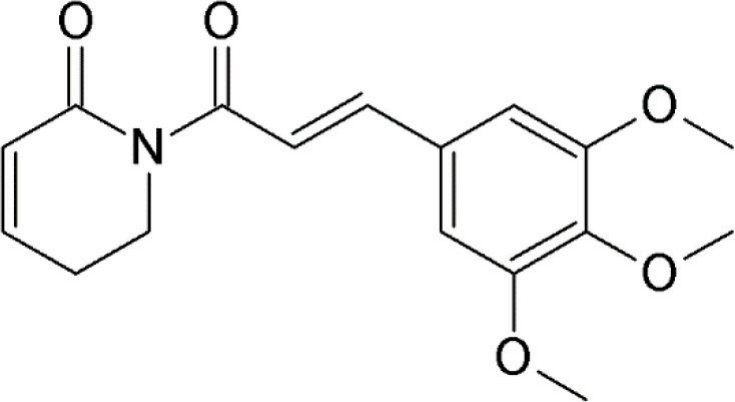	16.3 μM in HeLa cells.	Exhibits multiple biological and pharmacological activities including suppression of constitutive NFκB, AKT/mTOR and MAPK signaling pathways and induces apoptosis in tumorigenic cells.	Ginzburg et al., 2014; Shrivastava et al., 2014; Liu Q. R. et al., 2014; Niu et al., 2015
Plumbagin	*Plumbago zeylanica*	Covalently binds to cysteine528	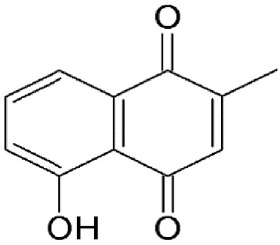	Inhibitory effects observed between 1 and 3 μM in tumor cell lines.	Retains FOXO1, p21, p53, and p73 in the nucleus and suppress constitutive NFκB and Bcl2 activity.	Ahmad et al., 2008; Liu X. et al., 2014
				Inhibits CRM1 between 10 and 15 μM *in vitro*.		
Curcumin	*Curcuma longa*	Covalently binds to cysteine528	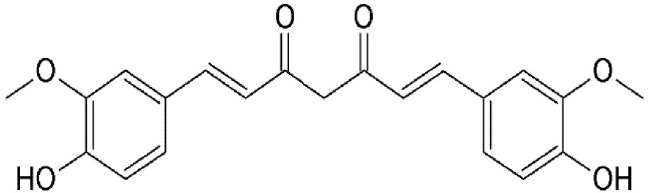	CRM1-inhibition at 100 μM.	Wide array of pharmacological properties including suppression of constitutive the NF-kB, MD2 and Akt/mTOR signaling pathways. Nuclear retention of FOXO1, p27 and p73.	Kim et al., 2003; Aggarwal and Shishodia, 2004; Gradisar et al., 2007; Niu et al., 2013
					Down regulation of proinflammatory proteins COX-2 and Cylin D1.	
15-Deoxy-Prostaglandin J2(15d-PGJ2)	Synthetic	Covalently binds to cysteine528	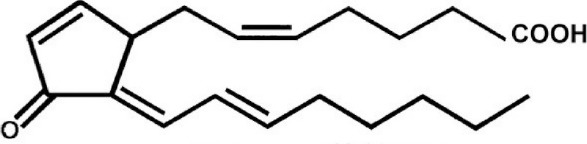	CRM1-inhibition between 10 and 30 μM for *in vivo* assays.	Inhibits the formation of CRM1-containing export complexes in both *in vitro* and *in vivo* assays.	Hilliard et al., 2010

#### N-azolylacrylate analogs

An N-azolylacrylate analog, PKF050-638 (Table 2) developed by Daelemans et al. was found to inhibit the nuclear export of HIV-1 Rev protein, a crucial factor for viral infection (as discussed in the previous section). PKF050-638 reversibly disrupts CRM1-NES interaction, targeting Cys539 in a dose-dependent manner at micromolar concentrations. PKF050-638 displayed high selectivity in binding to CRM1 while its trans-enantiomer (PKF050-637) was ineffective (Daelemans et al., 2002).

#### CBS9106

CBS9106 (Table 2) was found to reversibly inhibit CRM1-mediated export both *in vivo* and *in vitro*. A cell cycle phenotype-based protocol was used to determine its anti-tumor activity against various cancer cell lines including multiple myeloma and showed varied cytotoxicity depending on the cell line (IC50 ranging between 3 and 278 nM). The compound induced cell cycle arrest and apoptosis in cancer cells in a time- and dose-dependent manner in both *in vivo* and *in vitro* models. In multiple myeloma cells, reduced NF-κB activity due to CRM1-inhibition was found to be a key factor for the inhibitory effects of CBS9106 (Sakakibara et al., 2011). A commercially cost-effective derivative of CBS9106 designated S109 was also shown to reversibly inhibit CRM1-meidated nuclear export in renal cancer cells and reduce proliferation and colony formation. S109 down regulated the expression of cyclinD1, a nuclear protein that drives the G1 to S phase transition in the cell cycle, and induced the expression and accumulation of p53, p21, p27, and FOXO1. This resulted in cell cycle arrest at G1 phase in renal cancer cell lines (Liu et al., 2016).

Both CBS9106 and S109 were found to exert CRM1 inhibition similar to LMB. However, unlike LMB they induced a reduction in CRM1 protein levels without affecting CRM1 mRNA expression. CBS9106 is shown to induce proteasome-dependent CRM1 protein degradation since treatment with bortezomib counteracted this effect (Saito et al., 2014). S109 is also likely to have the same mechanism (Liu et al., 2016).

#### Selective inhibitors of nuclear export (SINE)

SINEs are first-in-class, novel selective inhibitors of nuclear export (KPT-SINE) developed by Karyopharm pharmaceuticals, USA using Concensus Induced Fit Docking (cFID). SINEs are designed such that they bind to CRM1 covalently, similar to LMB, but reversibly (Fung and Chook, 2014; Perwitasari et al., 2014). KPT-SINE compounds are orally bioavailable compounds developed as chemotherapeutics for various solid and hematologic malignancies (Turner and Sullivan, 2008; Turner et al., 2014; Sun et al., 2016). By exporting them from the nucleus of normal cells, CRM1 prevents multiple tumor suppressor proteins from acting in the absence of DNA damage or other oncogenic insults; thus, SINE compounds force the nuclear retention, accumulation, and functional activation of tumor suppressor proteins to limit oncogenesis (Perwitasari et al., 2014).

The administration of KPT compounds to animals is significantly less toxic than LMB (Fung and Chook, 2014). Two KPT SINE inhibitors (KPT 330 or Selinexor and KPT 335 or Verdinexor) are now in Phase 1/2 clinical trial for solid and hematological cancers (www.clinicaltrails.gov). SINE-mediated CRM1 inhibition enables nuclear retention of tumor suppressor proteins and anti-apoptotic signals (IκB, Survivin, p53, NPMc mutant, p27, and FOXO) and lead to cell cycle arrest and apoptosis in cancerous cells (Parikh et al., 2014; Das et al., 2015).

##### SINEs are slow-reversible inhibitors of CRM1

SINEs are typically 350 Da compounds, in comparison to 540Da LMB, sharing a phenyl triazole scaffold with different Michael addition acceptor side chains. The smaller structure of SINEs in comparison to LMB (Table 2) translates as 40% occupancy of the NES groove leaving the remaining portion of the hydrophobic groove open and unoccupied. SINEs bind to CRM1 with their trifluoromethyl groups buried deep within the NES binding groove. Unlike LMB, SINEs are not hydrolyzed after conjugation probably due to its attachment deep into the NES groove protected from potential nucleophiles and oxyanions. The conjugation of KPT 185 to CRM1 and its inhibition activity is reversed by 40–60% after 24 h most likely due to a lack of the hydrolysis of its active enone group. The slow reversibility of SINEs possibly contributes to their improved tolerance since withdrawal of the drug allows deconjugation of the drug from CRM1 and essential nuclear export to resume in normal cells. They bind long enough to kill cancer cells but their reversible nature allows then to be released in time to spare normal cells (Sun et al., 2013; Fung and Chook, 2014; Dickmanns et al., 2015).

## Other naturally occurring inhibitors

Several small molecules found in nature have CRM1 inhibitor activity; several such molecules are already in use in mainstream and/or complementary medicine with bioavailability and *in vivo* safety data already in hand. These present an attractive group of drugs for development of antiviral strategies.

Antiviral activities have been reported for numerous medicinal plants and have been extensively used as part of traditional medicine globally for centuries. Herbal products with confirmed clinical safety features are attractive starting material for the identification of new antiviral activities. Recent demonstration of anti-HIV-1 activity of extracts of *Pelargonium sidoides*, licensed in Germany as the herbal medicine Umckaloabo® suggests that investigation of antiviral activities among herbal extracts holds immense potential (Niu et al., 2013, 2015; Forouzanfar et al., 2014; Helfer et al., 2014; Liu X. et al., 2014; Wang et al., 2015). A selection of naturally occurring CRM1 inhibitors are described below.

### FOXO family export inhibitors

FOXO or Forkhead family of transcription factors (FOXO1a, FOXO3a, and FOXO4) are activated in response to tumor suppressors, such as PTEN lipid phosphatase to retard cell growth and induce apoptosis (Nakamura et al., 2000). Defective PTEN expression and/or CRM1 overexpression results in mislocalization of FOXO to the cytoplasm and promotes tumorigenesis (Ramaswamy et al., 1999; Brunet et al., 2002). In search for FOXO inhibitors Kau et al. (2003) utilized a cell-based, chemical genetic screening regimen to screen compounds from the NCI Structural Diversity Set, ChemBridge DiverSetE, and a small collection of NCI marine extracts to detect nuclear localization of FOXO. 11 compounds were found to interrupt CRM1-mediated nuclear export targeting Cys528. Eight of these compounds had an α,β-unsaturated ketone or amide group capable of Michael-type addition with Cys528 and the other 3 compounds probably modified Cys528 through a nucleophilic attack or induced a chemical rearrangement (Kau et al., 2003)

### Valtrate, acetoxychavicol acetate, and prenylcoumarin osthol

These are small molecule inhibitors of CRM1 isolated from *Valerianae fauriei, Alpinia galangal*, and *Cnidii Monnieris Fructus*, respectively (Table 2). All three compounds were shown to disrupt CRM1-mediated export of HIV-1 Rev protein. Valtrate and acetoxychavicol acetate have also been shown to inhibit nuclear export of influenza viral RNP (Watanabe et al., 2001, 2011). Their mechanism of action was demonstrated to be the same as LMB (Murakami et al., 2002; Tamura et al., 2010a,b).

### Ratjadone analogs

Ratjadone analogs A, B, C, and D isolated from a myxobacterium *Sorangium cellulosum* (Table 2), are CRM1 inhibitors that have anticancer and antifungal properties. Being similar in structure to LMB, ratjadones use the same molecular mechanism to inhibit CRM1 (Koster et al., 2003; Meissner et al., 2004). Drug resistant-human multiple myeloma cell lines became more sensitized after inhibition of CRM1-meidated nuclear export using ratjadone. They were more responsive to topoIIα inhibitors (such as doxorubicin and epoposide) and to anticancer drugs, such as topotectan (a topoisomerase inhibitor) and cis-platinum (a DNA cross linking agent) (Turner et al., 2009). Treatment with ratjadones has shown to inhibit cell proliferation by inducing cell cycle arrest at G1 and an increase in size of the nuclei (indicative of effective block of nuclear export) (Burzlaff et al., 2003; Koster et al., 2003). Recently ratjadone A has been shown to exert anti-HIV activity *in vitro* in a dose-dependent manner at nanomolar concentrations. Its inhibitory effect occurs 12 h.p.i. and interferes with the formation of the CRM1-Rev-NES complex by binding to CRM1 but not to Rev (Fleta-Soriano et al., 2014).

### Goniothalamin and anguinomycin

A styryl lactone isolated from *Goniothalamus* (*Annonaceae*), goniothalamin (Table 2) has a broad range of pharmacological properties including antimicrobial and anti-tumorigenic activity (Mosaddik and Haque, 2003; Seyed et al., 2014). Goniothalamin has been reported to induce cell cycle arrest at the G2/M phase and apoptosis in breast cancer cells with IC50 value of 1.46 μM (Chen et al., 2005). The compound was shown to inhibit CRM1-mediated nuclear export at 1 μM using the same mechanism as LMB (Wach et al., 2010).

Anguinomycin isolated as a natural product from *Streptomyces* spec. and produced by total chemical synthesis synthetized have been reported to shutdown CRM1-mediated nuclear protein export at concentrations above 10 nM (Dickmanns et al., 2015). Its α,β-unsaturated lactone group interacts with CRM1 in a similar mechanism to LMB (Bonazzi et al., 2010).

### Piperlongumine

Piperlongumine (5,6-dihydro-1-[(2E)-1-oxo-3-(3,4,5-trimethoxyphenyl)-2-propenyl]-2(1H)-pyridinone) is a natural alkaloid of the Long pepper (*Piper longum* L. – *Piperaceae*) widely used in Indian and Chinese traditional medicine (Table 2). Piperlongumine exhibits multiple biological and pharmacological activities including antimicrobial, anti-inflammatory, platelet aggregation inhibitor and antitumor activities; reviewed in Bezerra et al. (2013). The electrophilic α,β-unsaturated carbonyl group of piperlongumine has been shown to covalently modify Cys528 of CRM1 in a Michael addition manner and inactivate CRM1-mediated protein export (Niu et al., 2015). This suggests piperlongumine-induced suppression of constitutive NFκB, Akt/mammalian target of rapamycin (mTOR) and MAPK signal pathways and cytotoxicity in tumorigenic cells demonstrated in several studies could be explained by its interference with CRM1-mediated export (Ginzburg et al., 2014; Shrivastava et al., 2014; Liu Q. R. et al., 2014). Piperlongumine was demonstrated to inhibit CRM1-mediated nuclear export in HeLa cells after 24 h-exposure with an IC50 value of 16.3 μM, showing cellular tolerance and potential for oral administration (Niu et al., 2015).

### Plumbagin

Plumbagin, a natural bicyclic naphthoquinone (Table 2) derived from *Plumbago zeylanica*, is known to have antimicrobial, anticancer, antiproliferative, chemopreventive, chemotherapeutic, and radiosensitising properties (Ahmad et al., 2008; Aziz et al., 2008; Sinha et al., 2013). It is known to induce apoptosis and cell cycle arrest by suppressing constitutive the NF-kB signal pathway (Ahmad et al., 2008). Plumbagin has been demonstrated to interfere with CRM1-mediated export, directly interacting with the transporter protein same as LMB which could explain some of its therapeutic properties (Liu X. et al., 2014). Plumbagin disrupts CRM1 export and retains tumor suppressors, such as FOXO1, p21, p53, and p73 in the nucleus suggesting this as a mechanism for the naphthoquinone's antitumorigenic effects (Liu X. et al., 2014).

### Curcumin

Curcumin (Diferuloylmethane) is the major constituent of *Curcuma longa* (Table 2), an ancient spice widely used in Indian traditional medicine. Extensive research has showed curcumin as the major bioactive chemical responsible for a wide array of pharmacological properties that involve regulation of various cellular growth and transcription, cytokines production or inhibition, protein kinases, and other enzymes (reviewed in Aggarwal et al., 2007; Jagetia and Aggarwal, 2007). Suppression of constitutive NF-kB signal pathway (Aggarwal and Shishodia, 2004), myeloid differentiation protein 2 (MD-2) (Gradisar et al., 2007), Akt/ mTOR (Beevers et al., 2009), and STAT3 signaling (Kim et al., 2003) have been suggested for the antitumorigenic effects of curcumin. Recently, CRM1 has been confirmed as one of the cellular targets directly interacting with curcumin. Curcumin was shown to covalently interact with CRM1 at Cys528, induce the nuclear retention of FOXO1 and upregulates the expression of p73 and p27 in HeLa cells. In addition, CRM1 modulation by curcumin downregulated the expression of proinflammatory proteins COX-2 and cyclin D1 in a dose-dependent manner (Niu et al., 2013).

### 15-deoxy-prostaglandin J2(15d-PGJ2)

Prostaglandins (PGs) are signaling molecules involved in inflammation, hemostasis, gastrointestinal secretion, thrombosis, and other cellular functions. 15d-PGJ2 is a member of the prostaglandin (PG) J(2) family and has both anti- and pro-inflammatory properties. At low concentrations, endogenous 15d-PGJ2 exerts anti-inflammatory effects, promoting chemotaxis and activation of eosinophils, while at high concentrations it induces eosinophil apoptosis in a PPARγ-independent manner (Miwa et al., 2004; Ueki et al., 2007). Exogenous administration of 15d-PGJ_2_ has shown to have antiviral, antiproliferative and anti-inflammatory effects. 15d-PGJ_2_ promotes translocation of PPARγ to the nucleus and activation of NFκB/IκB signaling pathway to mediate anti-inflammatory responses. Exogenous 15d-PGJ_2_ (Table 2) has been shown to interrupt CRM1-mediated nuclear export by interacting with CRM1 similar to LMB (Hilliard et al., 2010). In comparison to LMB higher concentrations of 15d-PGJ_2_ are required to inhibit CRM1 probably because its α,β-unsaturated carbonyl group can only modify selected or exposed nucleophilic groups. However, this also enables targeted inhibition of a subset of proteins. 15d-PGJ_2_ and/or derivatives of this PG as a CRM1 inhibitor hold promise as therapeutic agents as it can be produced by higher eukaryotes and can therefore function as both endogenous and exogenous regulator of CRM1 mediated nuclear export (Hilliard et al., 2010).

## Potential natural CRM1 inhibitors

In addition to the above listed natural CRM1 inhibitors, several natural compounds have been demonstrated to inhibit viruses, interfering with the nuclear export of viral components, both protein and RNA. The similarity between the effect of LMB and some natural compounds present in traditional medicines suggest that they are CRM1-inhibitors. For instance, thymoquinone, the main constituent of the volatile oil from *Nigella sativa* (black cumin) seeds, is reported to protect laboratory animals against chemical toxicity and induction of carcinogenesis. Thymoquinone reacts *in vitro* with glutathione through a spontaneous and rapid reaction that produces a dihydrothymoquinone-thioether via 1,4-Michael addition mechanism, like LMB, thereby suggesting that thymoquinone may be capable of inhibiting CRM1 (Forouzanfar et al., 2014).

A lignan glycoside isolated from the latex of *Calotropis gigantea* (*Asclepiadaceae*), (+)-pinoresinol 4-O-[6″-O-vanilloyl]-β-d-glucopyranoside, has been shown to exert anti-influenza activity at the early stage of viral replication in both influenza A [A/PR/8/34 (H1N1), A/FM/1/47 (H1N1), and A/Aichi/2/68 (H3N2)] and influenza B (B/Lee/1940) subtypes. The lignan glycoside efficiently inhibited the virus-induced translocation of NF-κB and the activation of the NF-κB pathway in a dose-dependent manner (IC50 from 13.4 to 39.8 μM). The extract also reduced nuclear export of viral ribonucleoproteins, suggesting the involvement of CRM1 interaction (Parhira et al., 2014).

Clemastanin B, a lignan isolated from *Isatis indigotica* root, inhibits human [H1N1, H3N2, and influenza B (B/Guangzhou/GIRD08/09)] and avian influenza viruses (H6N2, H7N3, and H9N2) with IC50 ranging from 0.087 to 0.72 mg/ml. The anti-influenza compound was found to reduce viral titer in in infected MDCK cells at early stages of infection. Although the mechanism of action has not been elucidated, nuclear retention of viral ribonucleoproteins in the treated cells suggests clemastanin B targets viral endocytosis, uncoating or RNP export from the nucleus to exert its effects (Yang et al., 2013).

Licorice (*Glycyrrhiza uralensis* Fisch.) is a common herb used in traditional Chinese medicine for airway symptoms that have been proved to have anti-RSV activity (Chang et al., 2011, 2012; Wang et al., 2011). These prescriptions have been shown to exert antiviral properties by stimulating anti-viral cytokines, such as interferon-β and TNF-α in both upper (Hep-2) and lower (A549) respiratory epithelial cell lines (Feng Yeh et al., 2013). Glycyrrhizin (GL), 18β-glycyrrhetinic acid (GA), liquiritigenin (LTG), licochalcone A (LCA), licochalcone E (LCE), and glabridin (GLD) are the main active components in licorice extracts which possess antiviral and antimicrobial activities (Wang et al., 2015). GL and GA have been shown to inhibit replication of rotavirus, RSV, HIV etc. by inhibiting virus replication, preventing viral attachment or enhancing host cell activity. But their mechanism of action is yet to be investigated (Wang et al., 2015).

## Conclusion and future prospects

Regulated, appropriate translocation and subcellular localization of proteins is essential for regulation of replication, transcription and translation. Dysregulation of this system is observed in several pathological conditions including cancer and viral infections. Shuttling transport receptors mediate all cargo transport across the nuclear pore complex via similar mechanisms, ensuring regulated localization of transcription and growth factors, cytokines and other proteins/mRNA. CRM1 is a conserved and well characterized nuclear exporter that binds to protein/mRNA cargoes tagged with an NES motif. It is the sole exporter for several oncogenes and tumor suppressor proteins and is involved in regulating cell cycle, ribosome biogenesis and maintenance of chromosomal and nuclear structures.

Overexpression of CRM1 is observed in solid and hematologic malignancies. Mislocalization of regulatory factors away from their original site of action in the nucleus promotes malignancy, evasion of apoptosis and detection, and drug resistance. CRM1-mediated export is also co-opted by viruses belonging to diverse families at various stages of their replication to mediate infection and retard host antiviral responses. Disruption of CRM1-mediated nuclear export in both cancer and viral infections resulted in increased response to therapeutic agents and successful elimination of abnormal/infected cells.

Several small molecule inhibitors of CRM1 have been developed and tested against a variety of neoplastic cells and viral infected cell lines, primarily *in vitro*. LMB, the first and prototypical inhibitor, targets Cys528 of CRM1, altering its three-dimensional structure and capacity to transport cargo across the NPC. The high cytotoxicity of LMB makes it unsuitable for therapeutic purposes; however it initiated the search for synthetic and natural alternatives capable of eliciting the same effects without the cytotoxicity.

Synthetic CRM1 inhibitors, such as KOS2464, CBS9106, N-azoylacrylate analogs, and SINEs have proven capable of interrupting tumorigenesis in both solid and hematological malignancies. However, synthetic drugs present the risk of unwanted side effects and development of resistance in both cancer and viruses. Numerous medicinal plants have been extensively used as part of traditional medicine globally for centuries, several of these have been shown to have CRM1 inhibiting activity and exert antiviral properties. These present an attractive group of drugs for development of antiviral strategies since they are already in use in mainstream and/or complementary medicine with bioavailability and *in vivo* safety data already in hand; importantly, these compounds have relatively low cytotoxicity compared to synthetic CRM1 inhibitors.

## Author contributions

CM and RG have contributed equally to the conception and design of the work including drafting and revising the work critically for important intellectual content; both authors have approved the version to be published and agree to be accountable for all aspects of the work.

### Conflict of interest statement

The authors declare that the research was conducted in the absence of any commercial or financial relationships that could be construed as a potential conflict of interest.
